# In silico functional, structural and pathogenicity analysis of missense single nucleotide polymorphisms in human *MCM6* gene

**DOI:** 10.1038/s41598-024-62299-2

**Published:** 2024-05-21

**Authors:** Md. Mostafa Kamal, Md. Sohel Mia, Md. Omar Faruque, Md. Golam Rabby, Md. Numan Islam, Md. Enamul Kabir Talukder, Tanveer A. Wani, M. Atikur Rahman, Md. Mahmudul Hasan

**Affiliations:** 1 Department of Nutrition and Food Technology, Jashore University of Science and Technology, Jashore, 7408 Bangladesh; 2 Department of Food Engineering, North Pacific International University of Bangladesh, Dhaka, Bangladesh; 3 Laboratory of Computational Biology, Biological Solution Centre, Jashore, 7408 Bangladesh; 4 Department of Pharmaceutical Chemistry, College of Pharmacy, King Saud University, 11451 Riyadh, Saudi Arabia; 5 Department of Biological Sciences, Alabama State University, 915 S Jackson St, Montgomery, AL 36104 USA

**Keywords:** Single nucleotide polymorphisms, Missense SNPs, Pathogenicity prediction and computational tools, Computational biology and bioinformatics, Genetics

## Abstract

Single nucleotide polymorphisms (SNPs) are one of the most common determinants and potential biomarkers of human disease pathogenesis. SNPs could alter amino acid residues, leading to the loss of structural and functional integrity of the encoded protein. In humans, members of the minichromosome maintenance (MCM) family play a vital role in cell proliferation and have a significant impact on tumorigenesis. Among the MCM members, the molecular mechanism of how missense SNPs of minichromosome maintenance complex component 6 (MCM6) contribute to DNA replication and tumor pathogenesis is underexplored and needs to be elucidated. Hence, a series of sequence and structure-based computational tools were utilized to determine how mutations affect the corresponding MCM6 protein. From the dbSNP database, among 15,009 SNPs in the *MCM6* gene, 642 missense SNPs (4.28%), 291 synonymous SNPs (1.94%), and 12,500 intron SNPs (83.28%) were observed. Out of the 642 missense SNPs, 33 were found to be deleterious during the SIFT analysis. Among these, 11 missense SNPs (I123S, R207C, R222C, L449F, V456M, D463G, H556Y, R602H, R633W, R658C, and P815T) were found as deleterious, probably damaging, affective and disease-associated. Then, I123S, R207C, R222C, V456M, D463G, R602H, R633W, and R658C missense SNPs were found to be highly harmful. Six missense SNPs (I123S, R207C, V456M, D463G, R602H, and R633W) had the potential to destabilize the corresponding protein as predicted by DynaMut2. Interestingly, five high-risk mutations (I123S, V456M, D463G, R602H, and R633W) were distributed in two domains (PF00493 and PF14551). During molecular dynamics simulations analysis, consistent fluctuation in RMSD and RMSF values, high Rg and hydrogen bonds in mutant proteins compared to wild-type revealed that these mutations might alter the protein structure and stability of the corresponding protein. Hence, the results from the analyses guide the exploration of the mechanism by which these missense SNPs of the *MCM6* gene alter the structural integrity and functional properties of the protein, which could guide the identification of ways to minimize the harmful effects of these mutations in humans.

## Introduction

Single nucleotide polymorphisms (SNPs) are the most widespread and reliable kind of genetic variation that is associated with disease development and guide the exploration of the mechanisms of disease pathogenesis. The human genome contains numerous genetic code variations, with SNPs being the most abundant, accounting for nearly 1% of the entire human genome^1^. These SNP-mediated variations alter the genomic sequence by changing the intergenic region (regions between genes), coding region of genes (exons), and non-coding region of genes (introns)^2^. SNPs in the coding region are divided into two types, synonymous and non-synonymous SNPs (nsSNPs), where protein sequences are altered by the nsSNPs^3^. Synonymous mutations cause no change in the corresponding protein due to the degenerative alternative code of the amino acids. Although having a potential impact on the splicing process, these synonymous SNPs (sSNPs) are considered functionally inactive and have negligible impact on evolutionary processes^4^. Indeed, among the nsSNPs, missense SNPs mainly change the structure, stability, and functions of the corresponding protein^5^. Since intronic regions do not participate in translation, the SNPs in the region have the least contribution to disease pathogenesis. Due to representing approximately half of the human non-coding genome, introns contribute greatly to genome evolution^6^.

Transcription factors play a vital role in the pathogenesis of human diseases. Among the transcription factors in the human genome, members of the minichromosome maintenance complex (MCM) gene family play a vital role in cell proliferation and have a potential impact on tumorigenesis^7^. The MCM family includes MCM2, MCM3, MCM4, MCM5, MCM6 and MCM7 protein complexes. The *MCM2* gene plays a vital role in DNA replication and overexpression is associated with multiple types of cancers^8^. The *MCM3* gene is essential for the initiation of DNA replication and is involved in ensuring the precise initiation of DNA replication once per cell cycle^9^. The *MCM4* gene is mainly enriched in the cell cycle and cell division and is also significantly associated with tumor size and, lymph node metastasis^10^. The *MCM5* gene is associated with malignant status and poor prognosis in cervical adenocarcinoma patients, modulates cervical adenocarcinoma cells proliferation, inhibits the cell cycle and promotes colorectal cancer cells in vitro^11,12^. The *MCM6* gene is located at 2q21^13,^ spans 3,624 bp and encodes 821 amino acids with a molecular weight of 93.1 kDa^14^. It plays a significant role in the regulation of DNA replication by forming a hetero-hexameric complex with other MCM members^15^. In addition, the *MCM6* gene is involved in tumor pathogenesis^16^ and promotes the progression of hepatocellular carcinoma (HCC)^14^. It also plays a vital role in cell proliferation, migration, invasion and the immune response in many cancer types, such as breast cancer^17,18^, HCC^16^, glioma^19^, esophageal squamous cell carcinoma (ESCC)^20^ and endometrioid endometrial adenocarcinoma^21^. The *MCM7* gene plays a significant role in eukaryotic DNA replication, and its overexpression is related to cellular proliferation and responsible for various cancers^22^.

SNPs are the widespread genetic variation that includes missense SNPs, which are associated with and act as biomarkers of disease pathogenesis by affecting gene function^23^. Deleterious missense SNPs that have the potential to destabilize proteins, significantly affects protein structure upon a single amino acid substitution, disease-association, be highly conserved could be a potential biomarker for specific diseases^24^. The missense SNPs in the *MCM6* gene could disrupt the binding ability of the respective proteins. Non-synonymous variants of *MCM6* gene are associated with lactase persistence in Africans and Europeans^25^. It also has a homozygous mutation in *ELMO*3 gene, which is associated with Keratoconus^26^. The continuous incursion of new variants in different genes can easily be tracked using modern molecular biology techniques. However, the molecular mechanisms by which MCM6 variants contribute to disease pathogenesis in humans are yet to be discovered. Considering the above-mentioned facts, we performed extensive screening for the most damaging missense SNPs in *MCM6* gene to identify the pathogenic SNPs. The *MCM6* gene was extracted from the NCBI database and screened for high-risk pathogenicity using multiple bioinformatics tools with the highest precision level^27–29^. In addition, the mechanism by which pathogenic missense SNPs alter protein structure and function was also explored. Then, molecular dynamics (MD) simulation was also conducted to check the stability of the missense SNPs^30^.

## Materials and methods

**Figure 1:**
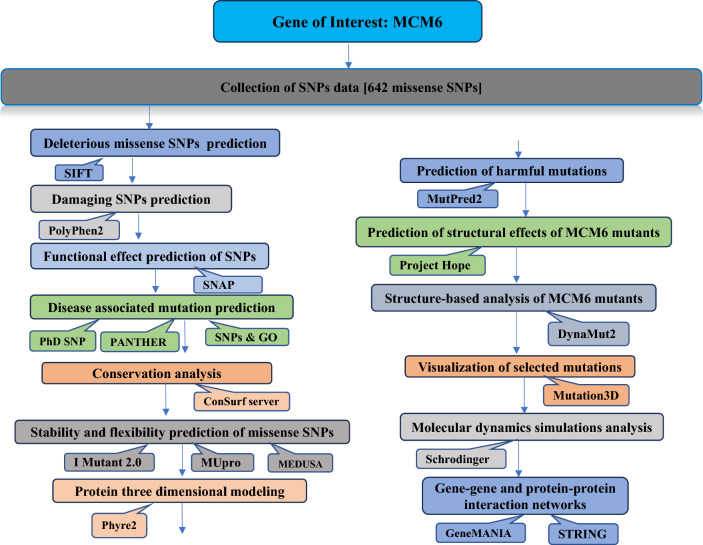
Schematic representation of the pipeline for in silico analysis of missense SNPs in the *MCM6* gene.

The schematic diagram of the in-silico analyses conducted in the study is presented in Fig. 1.

### Protein sequence and missense SNPs retrieval

National Center for Biotechnology Information (NCBI) (https://www.ncbi.nlm.nih.gov/) and NCBI dbSNP (https://www.ncbi.nlm.nih.gov/snp/) databases were used to collect the protein sequence (FASTA format) and SNPs of *MCM6* gene respectively. The missense SNPs were further analyzed using different software, as this type of mutation generates protein variants and induces crucial structural alterations that could decrease binding affinity and impair the protein function^31^.

### Deleterious missense SNPs prediction using SIFT

Sorting Intolerant from Tolerant (SIFT)^32^ (https://sift.bii.a-star.edu.sg/) is a bioinformatic web server used to detect deleterious missense SNPs from tolerated SNPs. This is a homology-based sequence analysis that calculates the normalized probabilities for all possible substitutions from the alignment. In SIFT prediction, missense SNPs having score of > 0.05 regarded as ‘tolerated’ and less than or equal of that regarded as deleterious.

### Damaging missense SNPs prediction using PolyPhen-2

Polymorphism Phenotypingv2 (PolyPhen-2)^33^ web server (http://genetics.bwh.harvard.edu/pph2/) is used to predict the possible impact of missense SNPs on protein structure and function. The analysis is based on the sequence, structure and phylogenetic relationships. PolyPhen-2 categorizes SNPs into three categories, (1) benign (0.00–0.45) (2) possibly damaging (0.45–0.95), and (3) probably damaging (0.95–1). The input FASTA sequence of the protein with the position of interest and the new residue were submitted to PolyPhen-2 to predict the functional impact of mutations.

### Functional effect prediction of missense SNPs using SNAP

Screening for non-acceptable polymorphisms (SNAP)^34^ (http://www.rostlab.org/services/SNAP) is a bioinformatics web server used to evaluate the functional effects of a single amino acid substitution in proteins using the neural network method. It predicts the changes that occur due to the missense SNPs on the secondary structure and compares the solvent accessibility of the native and mutated proteins to distinguish them into effect or neutral. The FASTA sequence of the native MCM6 protein was used as the input.

### Disease association prediction of missense SNPs

#### PhD-SNP

The predictor of human deleterious single nucleotide polymorphism (PhD-SNP)^35^ (http://snps.biofold.org/phd-snp/phd-snp.html) is a web server. It is based on Support Vector Machine (SVM) that is optimized to predict disease-related or neutral variants. FASTA sequences of the corresponding proteins and residue changes were submitted as inputs in the PhD-SNP server.

#### PANTHER

The protein analysis through evolutionary relationship (PANTHER)^36^ (https://pantherdb.org/) based web server was performed to evaluate the effect of the specific amino acid substitution in the biological function of the corresponding protein in the organism. Based on Hidden Markov Models (HMM), this server estimates the probability of how SNPs variants affect the structure of proteins based on their evolutionary origin.

#### SNPs&GO

The SNPs&GO^37^ server (http://snps-and-go.biocomp.unibo.it/snps-andgo/) also utilizes an SVM-based method that precisely predicts if the variants are disease-associated or not. This method calculates the score and evaluates the association of each mutated variant with human diseases. If the score of missense SNPs was ≥ 0.5, it was considered to be involved in the disease, while a score of < 0.5 was considered to have a neutral effect.

### Conservation analysis

The ConSurf^38^ (http://consurf.tau.ac.il/) web server detected a highly conserved functional network of the query protein. This tool creates a phylogenetic tree between homologous sequences to calculate the evolutionary conservation of the amino acids in a protein molecule.

### Stability and flexibility prediction of missense SNPs on MCM6 protein

#### I-Mutant2.0

The I-Mutant2.0^39^ web server (https://folding.biofold.org/cgi-bin/i-mutant2.0.cgi) was used to estimate the potential effects of missense SNPs on the structural reliability of the protein and free energy change DDG (Delta Delta G). This is an SVM-based prediction of changes in protein stability upon mutations in the corresponding protein. Here, stability increases when DDG is > 0 kcal/mol and decreases when DDG is < 0 kcal/mol.

#### MUpro

The MUpro^40^ server (http://mupro.proteomics.ics.uci.edu/) was used to predict the energy change and how mutations affect protein stability using both SVM and Neural Networks methods. A decrease in protein stability was predicted if the confidence score was < 0 while an increase in protein stability was predicted for a score of > 0.

#### MEDUSA

MEDUSA^41^ (https://www.dsimb.inserm.fr/MEDUSA/) web server was used to predict the flexibility of the corresponding protein. This provides a clear visualization of the prediction results. It predicts two, three and five classes of flexibility by using amino acid sequences. Following the evolutionary origin and physicochemical properties, the server categorized the flexibility class of each amino acid in the spatial arrangement of the protein. The amino acid sequence was put onto the server in FASTA format to obtain the results.

### Protein three-dimensional modeling

The Protein Homology/analogy Recognition Engine V 2.0 (Phyre2)^42^ web server (http://www.sbg.bio.ic.ac.uk/phyre2) was used to generate the three-dimensional (3D) structure of representative MCM6 and other mutant proteins. The FASTA sequences of the wild-type (WT) and other MCM6 mutant proteins were used to generate 3D structures^43^. The PyMOL^44^ software was used to visualize the homology models.

### Prediction of harmful mutations using MutPred2

MutPred2^45^ (http://mutpred2.mutdb.org/) is a web server that explains the reasons for diseases at the molecular level based on amino acid submissions. It predicts the molecular cause of a disease using a general probability score based on the gain/loss of 14 different structural and functional properties. This score represents the probability that an amino acid substitution is associated with a disease, and the top 5 property scores are provided, where p represents the p-value that certain structural and functional properties are impacted.

### Prediction of structural effects of MCM6 mutants using Project Hope server

Project Hope^46^ server (http://www.cmbi.ru.nl/hope/) was used to calculate the structural and functional effects of point mutations. This investigation provides 3D structural visualization of mutated proteins and provides the results using the UniProt and DAS prediction servers. Here, the protein sequence, wild-type, and new amino acids were used as inputs and the output resulted in text, graphics, and animation format.

### Structure-based analysis of mutations using DynaMut2

DynaMut2^47^ (https://biosig.lab.uq.edu.au/dynamut2/) was used to evaluate the mutations in protein stability and dynamics using the normal mode analysis (NMA) method. The predicted Gibbs free energy (ΔΔG) values of mutants less than zero (0) were classified as destabilizing, whereas those greater than 0 were classified as stabilizing.

### Visualization of selected mutations using mutation3D server

The mutation3D^48^ server (http://mutation3d.org/) is a functional prediction and visualization tool for studying the spatial arrangement of amino acid substitutions (AAS) in protein models and structures. This server was used to identify the clusters of amino acid substitutions using the 3D clustering method. It is also useful for clustering other kinds of mutational data, or simply as a tool to quickly assess the relative locations of amino acids in proteins. Additionally, it can be employed to cluster other types of mutational data or as a tool to quickly assess the relative locations of amino acids in proteins.

### Molecular dynamics simulations analysis

To evaluate the structural stability of the mutant protein, a 50 ns molecular dynamics (MD) simulation was performed using the "Desmond v6.3 Program" in Schrodinger 2020-3 under the Linux framework^49^. The simulation was performed following the three-site transferrable intermolecular potential (TIP3P) water model^50^. An orthorhombic box shape with a 10 Å distance from the center was used to maintain a specific volume, and Na^+^ and Cl^-^ were added to neutralize the whole system with a salt concentration of 0.15 M. An OPLS3e force field was applied^51^. The protein structure system was further minimized using a natural time and pressure (NPT) ensemble at a constant pressure of 1,01,325 Pascal’s and a temperature of 300 K. To evaluate the stability and dynamic characteristics of the protein, RMSD (root means square deviation), RMSF (root means square fluctuation), Rg (radius of gyration), and hydrogen bonds were analyzed.

### Gene–gene and protein–protein interaction networks

#### GeneMANIA

Gene–gene interaction network was used to understand the disease phenomenon. The GeneMANIA^52^ tool (http://www.genemania.org) predicts the biological function of a single gene or gene set and can help identify new genes in a pathway or complex. The human MCM6 protein sequence was used as input in GeneMANIA. The analyzed results were based on genetic interactions, pathways, co-expression, co-localization, and shared protein domain similarity.

#### STRING

Search Tool for the Retrieval of Interacting Genes (STRING)^53^ tool (https://string-db.org/) was used to identify the protein–protein interaction (PPI) of the MCM6 protein with other proteins in the human genome. The PPI network showed correlations between proteins. The PPI network and functional analysis indicated that protein sets were enriched in the target network of the MCM6 protein.

## Results

### Protein sequence and missense SNPs retrieval

The nsSNPs and sequence of the human *MCM6* gene were retrieved from the NCBI database. A total of 15,009 SNPs were identified for the *MCM6* gene. The automated computation resulted in 642 missense SNPs (4.28%), 291 synonymous SNPs (1.94%), and 12,500 intron SNPs (83.28%). Then, missense SNPs were further analyzed to identify the most deleterious variants.

### Deleterious missense SNPs prediction using SIFT

Among 642 missense SNPs, 33 SNPs were predicted to be deleterious with a tolerance index of ≤ 0.05 (Table 1 and Supplementary Table S1).

**Table 1:** Characteristics of missense SNPs in *MCM6* as predicted by different bioinformatic analyzes.

SL	SNP ID	Amino acid variants	SIFT	Polyphen2		SNAP	PhD-SNP	PANTHER	SNPs&GO
				HumDiv	HumVar				
1	rs201187605*	I123S	D	Prob.D	Prob.D	effect	Dis	Dis	Dis
2	rs201824504*	R207C	D	Prob.D	Prob.D	effect	Dis	Dis	Dis
3	rs144893830*	R222C	D	Prob.D	Prob.D	effect	Dis	Dis	Dis
4	rs374456478	V250I	D	Prob.D	Prob.D	effect	N	N	N
5	rs200725312	L284F	D	Prob.D	Prob.D	effect	N	NA	N
6	rs144937311	T357I	D	Prob.D	Prob.D	effect	N	N	N
7	rs368811703	T384N	D	Prob.D	Prob.D	effect	N	Dis	N
8	rs113753889	E438G	D	Prob.D	Prob.D	effect	N	Dis	N
9	rs376760086	I444M	D	Prob.D	Prob.D	effect	N	Dis	Dis
10	rs375104649*	L449F	D	Prob.D	Prob.D	effect	Dis	Dis	Dis
11	rs191517067*	V456M	D	Prob.D	Prob.D	effect	Dis	Dis	Dis
12	rs199696245*	D463G	D	Prob.D	Prob.D	effect	Dis	Dis	Dis
13	rs138808270	R468W	D	Prob.D	Prob.D	effect	Dis	Dis	N
14	rs377587920	Q470R	D	Prob.D	Prob.D	effect	N	N	N
15	rs201501566	I482V	D	Prob.D	Prob.D	effect	N	Dis	N
16	rs149081066*	H556Y	D	Prob.D	Prob.D	effect	Dis	Dis	Dis
17	rs184578188*	R602H	D	Prob.D	Prob.D	effect	Dis	Dis	Dis
18	rs374533979	D605G	D	Prob.D	Prob.D	effect	N	Dis	N
19	rs267598892*	R633W	D	Prob.D	Prob.D	effect	Dis	Dis	Dis
20	rs55828049	E647V	D	Prob.D	Prob.D	effect	Dis	N	N
21	rs373818867	I656V	D	Prob.D	Prob.D	effect	N	N	N
22	rs142938887*	R658C	D	Prob.D	Prob.D	effect	Dis	Dis	Dis
23	rs1804609*	P815T	D	Prob.D	Prob.D	effect	Dis	Dis	Dis

*More damaging SNPs (SNPs found to have functional significance by the 6 programs). D: Deleterious, E: Effect N: neutral, Dis: Disease, Prob.D: Probably damaging (more confident), NA: Unclassified.

### Damaging missense SNPs prediction using PolyPhen-2

Based on PolyPhen-2 analysis, 27 and 23 missense SNPs were observed as probably damaging with high confidence in HumDiv and HumVar analyses respectively. Subsequently, 23 were overlapped in both HumDiv and HumVar analyses and were considered for downstream experiments (Table 1).

### Functional effect prediction of missense SNPs using SNAP

Analysis of the 23 missense SNPs using SNAP program revealed that all inputted missense SNPs showed a significant effect. However, none of these SNPs was found to be neutral in this analysis (Table 1).

### Disease association prediction of missense SNPs

A total of 12, 13 and 17 SNPs were found to be associated with diseases when analyzed using the SNPs&GO, PhD-SNP, and PANTHER programs, respectively. Following all upstream analyses, 11 missense SNPs (I123S, R207C, R222C, L449F, V456M, D463G, H556Y, R602H, R633W, R658C, and P815T) were common and were observed as deleterious, probably damaging, affective and disease-associated (Table 1 and Supplementary Table S2).

### Conservation analysis

**Figure 2:**
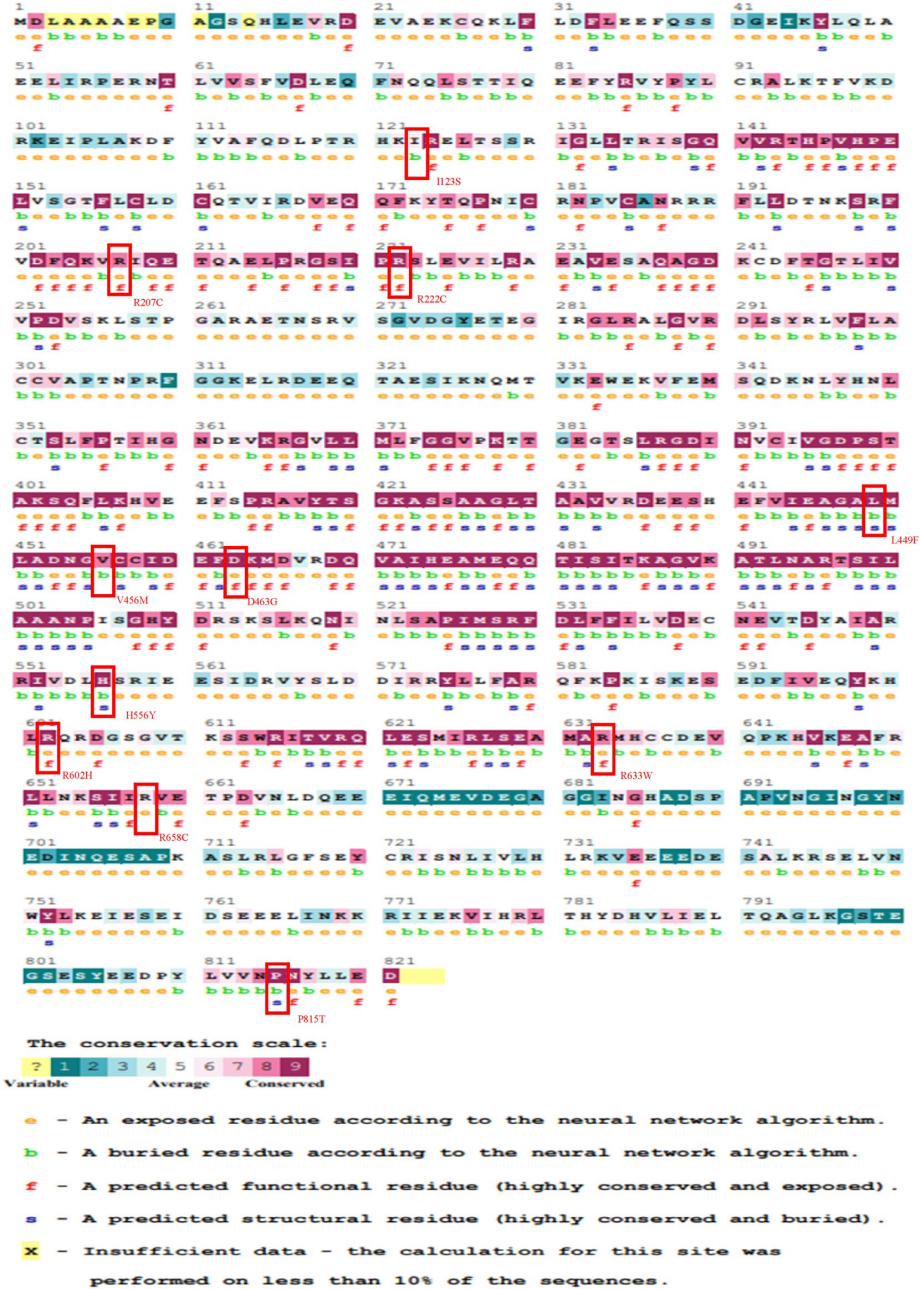
Analysis of evolutionary conserved amino acids residues of MCM6 by ConSurf. Color coding bar showing conservation score.

Specific positions of amino acids are crucial for the correct function of a protein. The ConSurf tool was used to determine the conservation score of the MCM6 protein. This program identified highly conserved structural and functional amino acid regions essential for biological functions. The analysis revealed that residues R222C, L449F, D463G, H556Y, R602H and P815T with a conservation score of 9, R207C, V456M, and R633W with a conservation score of 8, and I123S and R658C with a conservation score of 5 had potential biological functions. Interestingly, among those missense SNPs, R222C, R222C, D463G, R602H, and R633W were functional and the rest were buried (Fig. 2).

### Stability and flexibility prediction of missense SNPs on MCM6 protein

I-Mutant2.0 and MUpro servers were used to estimate the stability of 11 mutant proteins based on the free energy change value (Delta Delta G, DDG), and the confidence score respectively. Among these, 8 SNPs (I123S, R207C, R222C, V456M, D463G, R602H, R633W, and R658C) guided proteins were found to be the most unstable considering DDG and confidence score (Table 2).

**Table 2:** Characterization of the effect of missense SNPs on protein stability.

SL	Variant	I-Mutant2.0		MUpro			
		Stability	DDG	Stability	DDG	Stability	Confidence score
1	I123S	Decrease	− 0.93	Decrease	− 1.54802	Decrease	− 0.90178
2	R207C	Decrease	− 0.92	Decrease	− 0.59005	Decrease	− 0.76372
3	R222C	Decrease	− 0.42	Decrease	− 0.58448	Decrease	− 0.19823
4	L449F	Increase	1.27	Decrease	− 0.72186	Decrease	− 1
5	V456M	Decrease	− 1.59	Decrease	− 0.83729	Decrease	− 1
6	D463G	Decrease	− 1.52	Decrease	− 1.88655	Decrease	− 1
7	H556Y	Decrease	− 0.84	Decrease	− 0.49101	Increase	0.521393
8	R602H	Decrease	− 0.75	Decrease	− 1.11778	Decrease	− 0.82415
9	R633W	Decrease	− 0.06	Decrease	− 0.88862	Decrease	− 1
10	R658C	Decrease	− 0.51	Decrease	− 0.97542	Decrease	− 0.54781
11	P815T	Increase	1.85	Decrease	− 0.88742	Decrease	− 0.79865

**Figure 3:**
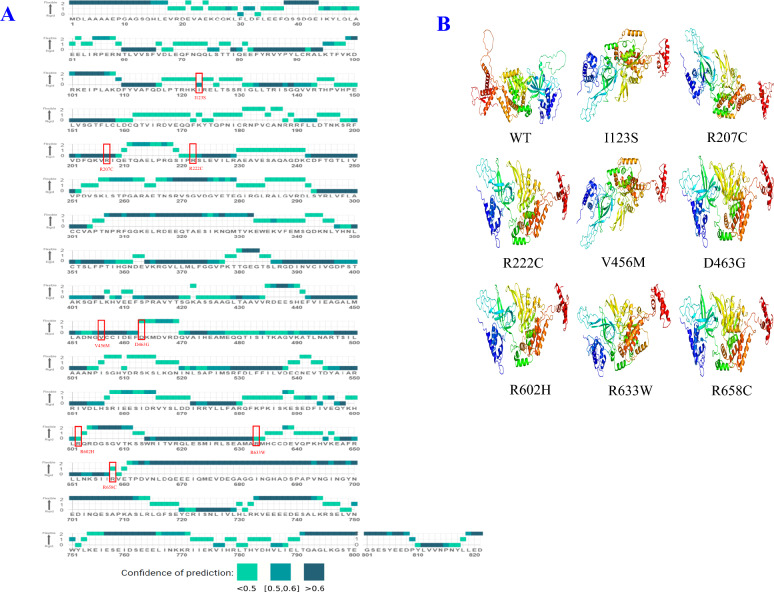
(**A**) Flexibility prediction of missense SNPs on MCM6 protein, (**B**) Three-dimensional modeling of wild type MCM6 protein and other 8 mutants. The protein chains are coloured as blue at the N-terminus and red at the C-terminus. The secondary structures in the model are highlighted in green (for α-helices) and yellow (for β-strands).

The MEDUSA web server predicted and visualized the flexibility of corresponding proteins with dynamic properties. Based on the three-class flexibility prediction of MCM6 protein by MEDUSA (0 = rigid, 2 = flexible), the positions of all 8 SNPs were rigid, except D463G and R658C which were flexible. The amino acid sequence positions R207C and V456M had a confidence score > 0.5, while the position I123S had the confidence score of 0.5–0.6 and positions R222C, D463G, R602H, R633W and R658C had a confidence score < 0.5 (Fig. 3A).

### Protein three-dimensional modeling

The Phyre2 homology-based modeling tool provided the 3D structure of the MCM6 WT and 8 mutant MCM6 proteins (Fig. 3B)^54^. Both WT and mutant proteins showed 100% confidence. The WT and mutant proteins showed 83% coverage of the corresponding proteins. The percentage of the alpha-helix in the MCM6 WT was 29%, whereas it varied from 29 to 31% in mutant proteins. Similarly, the beta strand in the MCM6 WT was 18%, whereas in the mutant protein it varied from 17 to 18%. Similarly, the disorder percentage varied from 22 to 24% (Fig. 3B and Supplementary Table S3).

### Prediction of harmful mutations by MutPred2

The MutPred2 tool provides the structural and functional effects of a specific protein based on its physiochemical properties. The eight potentially destabilized missense SNPs caused significant variations in the structural and functional properties of the corresponding MCM6 mutated proteins. Interestingly, these potential SNPs significantly promoted disease pathogenesis by altering various aspects of the protein, including helices and interfaces, DNA binding sites, allosteric sites, catalytic sites, pyrrolidone carboxylic acid, methylation, and transmembrane proteins. Finally, all 8 missense SNPs (I123S, R207C, R222C, V456M, D463G, R602H, R633W, and R658C) resulted in highly harmful mutated proteins, as indicated by the MutPred2 general score (Table 3).

**Table 3:** Prediction of pathogenicity of missense SNPs in MCM6 protein as predicted by MutPred2.

SL	Amino acid variants	MutPred2 score	Molecular mechanisms	P-value
1	I123S	0.937	Gain of Intrinsic disorder	0.02
			Gain of Allosteric site at R124	0.01
			Altered Transmembrane protein	0.02
2	R207C	0.883	Altered Ordered interface	0.02
			Altered Metal binding	0.04
			Loss of Acetylation at K205	0.04
3	R222C	0.915	Loss of Allosteric site at R222	0.0009
			Altered Ordered interface	0.0081
			Gain of Loop	0.0022
4	V456M	0.872	Altered Metal binding	0.0034
			Altered Ordered interface	0.04
			Loss of Relative solvent accessibility	0.03
			Loss of Allosteric site at E461	0.01
			Loss of Catalytic site at E461	0.01
			Altered Transmembrane protein	0.01
5	D463G	0.938	Altered Metal binding	0.00021
			Altered Ordered interface	0.05
			Loss of Allosteric site at D463	0.0075
			Loss of Relative solvent accessibility	0.03
			Gain of Catalytic site at E461	0.0066
			Gain of Acetylation at K464	0.03
6	R602H	0.892	Loss of Acetylation at K599	0.03
			Altered Disordered interface	0.04
			Altered DNA binding	0.03
7	R633W	0.876	Altered Metal binding	0.05
			Gain of Allosteric site at E629	0.03
8	R658C	0.842	Loss of Phosphorylation at T661	0.02
			Gain of Loop	0.03
			Loss of Acetylation at K654	0.0079
			Loss of ADP-ribosylation at R658	0.05
			Altered Transmembrane protein	0.0077

### Prediction of structural effects of MCM6 mutants using Project Hope server

The Project HOPE server showed how mutation affects the structural variation of a protein in terms of size, charge, hydrophobicity, and spatial structure compared to the wild type. Among the 8 predicted mutants, the I123S, R207C, R222C, D463G, R602H, and R658C mutant residues were smaller than the wild residues, which caused an empty space in the core of the wild protein and a loss of hydrophobic interactions. However, the V456M and R633W mutants were found to be larger in size than the wild residues, resulting in their localization on the surface of the wild protein. Therefore, mutations of residues can interrupt the inter/intra-molecular interactions of the protein (Table 4).

**Table 4:**
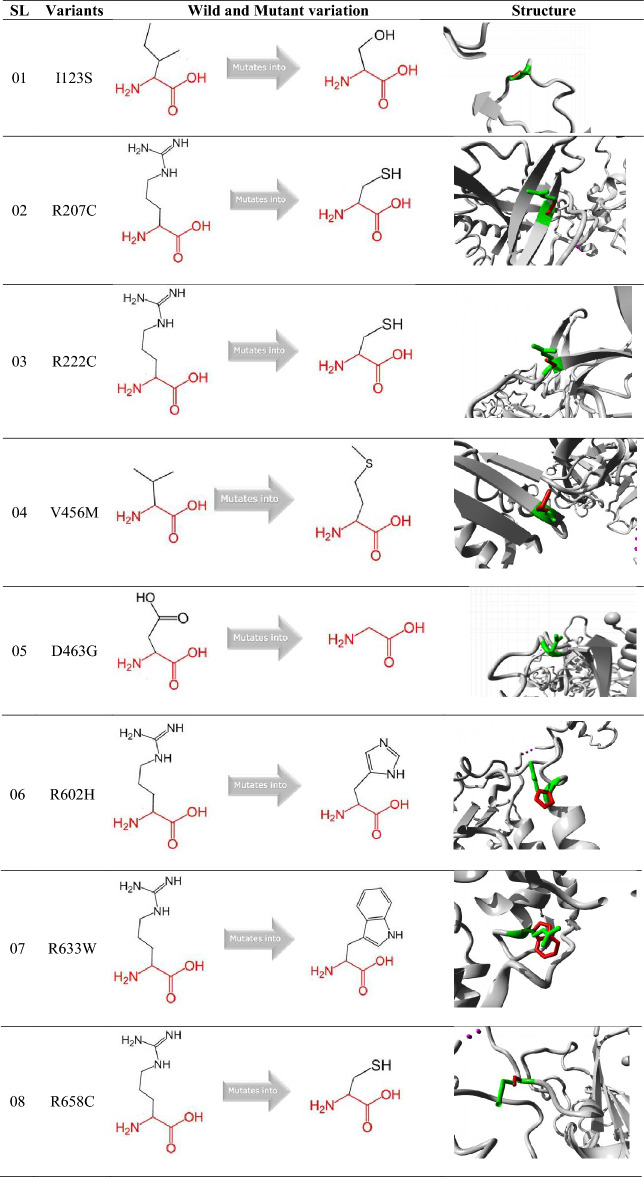
Prediction of how mutation affects MCM6 protein structure using Project Hope server.

Grey color indicates the chain of protein, Green colored atoms are the wild amino acid residues, and red are the mutated amino acid residues.

### Structure-based analysis of mutations using DynaMut2

The DynaMut2 server was used for mutation verification and its related effects on the spike protein structure and dynamics. Out of eight mutations, six (I123S, R207C, V456M, D463G, R602H, and R633W) mutations were found to be responsible for destabilizing the protein, whereas the remaining mutants were found to stabilize the protein structure (Table 5).

**Table 5:** Stability prediction of six most significant mutant proteins using DynaMut2.

SL	Variant	ΔΔG (kcal/mol)	Stability	Wild Type	Mutant
01	I123S	− 2.39	Destabilising		
02	R207C	− 0.69	Destabilising		
03	V456M	− 0.67	Destabilising		
04	D463G	− 0.31	Destabilising		
05	R602H	− 1.13	Destabilising		
06	R633W	− 1.6	Destabilising		

### Visualization of selected mutations using mutation3D server

**Figure 4:**
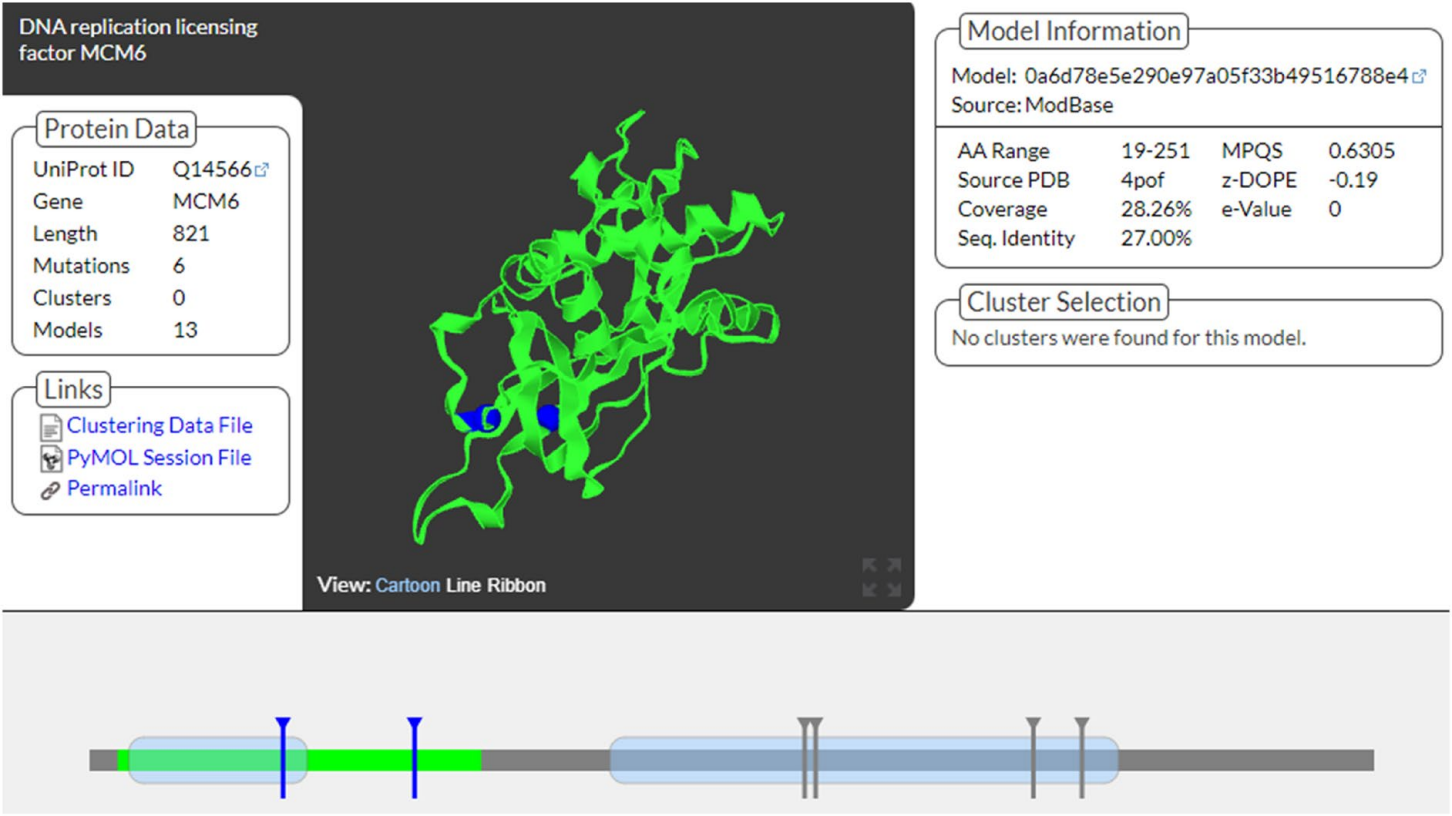
3D structure of MCM6 protein, generated by mutation 3D server, and representation of selected mutations on protein’s domains. Mutation3D^48^ server was used to generate the figure (http://mutation3d.org/).

The mutation3D server showed the presence of harmful substitutions in the MCM6 protein (encoded by the MCM6 gene), and the DNA replication licensing factor (Fig. 4). Two domains of the MCM6 protein, consisting of 821 amino acids were identified, MCM (PF00493) and MCM_N (PF14551). Among the six mutations, five (I123S, V456M, D463G, R602H, and R633W) were located in the domain region and were thus considered high-risk mutations for the MCM6 protein (Fig. 4).

### Molecular dynamics simulations analysis

#### RMSD

**Figure 5:**
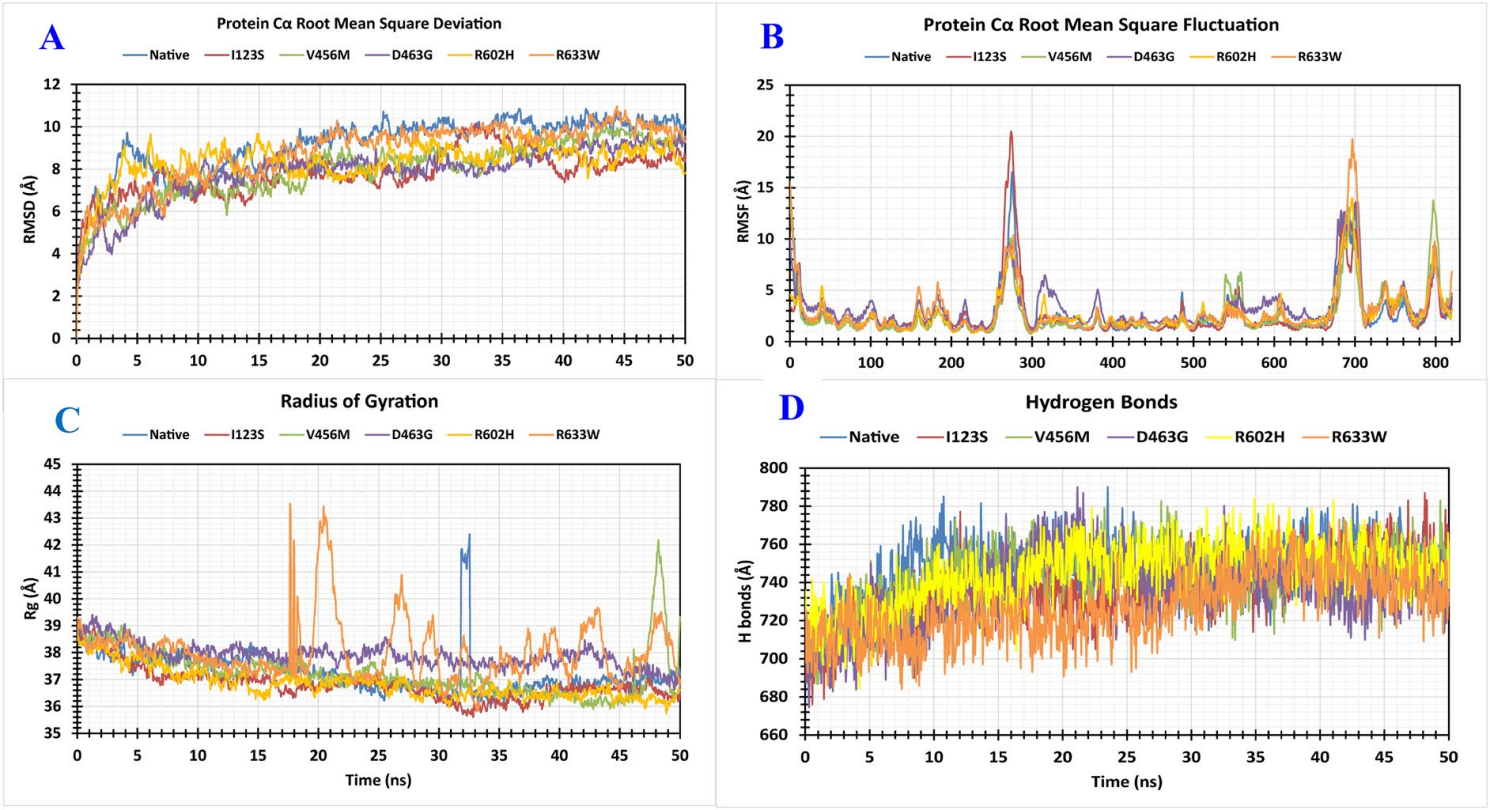
Analysis of RMSD, RMSF, Rg, and H bonds of native and five mutants of MCM6 over 50 ns simulation. (**A**) RMSD values of Cα atoms of native and mutant structures. (**B**) RMSF values of Cα atoms of native and mutant structures. (**C**) Calculation of Rg, which is represented as a time-dependent change during the simulation. (**D**) Calculation of H-bonds represented as a time-depend. The color scheme is as follows: native (blue), I123S mutant (red), V456M mutant (green), D463G mutant (violet), R602H mutant (yellow), and R633W mutant (orange). A 50 ns molecular dynamics (MD) simulation was performed using the "Desmond v6.3 Program" in Schrodinger 2020-3 under the Linux framework^49^. The simulation was performed following the three-site transferrable intermolecular potential (TIP3P) water model^50^. An orthorhombic box shape with a 10 Å distance from the center was used to maintain a specific volume, and Na^+^ and Cl^-^ were added to neutralize the whole system with a salt concentration of 0.15 M. An OPLS3e force field was applied^51^.

The RMSD of Cα atoms was subtracted for these wild-type and five mutant proteins to measure the protein structure stability throughout the 50 ns simulation. The wild protein and five selected missense SNPs proteins I123S, V456M, D463G, R602H, and R633W showed average fluctuations of 9.15 Å, 7.83 Å, 7.96 Å, 7.81 Å, 8.34 Å and 8.76 Å, respectively (Fig. 5A). This indicated that all the mutant proteins deviated similarly to the wild-type proteins. In the above proteins, the highest RMSD deviations were observed as 10.847 Å, 10.15 Å, 10.04 Å, 9.75 Å, 10.07 Å, and 10.95 Å and the lowest in the same were 2.147 Å, 2.027 Å, 1.813 Å, 2.254 Å, 1.915 Å and 1.844 Å respectively (Fig. 5A). Hence, mutated proteins had structural instability compared to the wild-type proteins.

#### RMSF

To investigate the variations in structural flexibility of specific amino acids in the proteins, the RMSF values were assessed (Fig. 5B). Wild-type and five mutated proteins I123S, V456M, D463G, R602H, and R633W had the highest peak fluctuations positioned at GLY_275, TYR_276, GLU_277, ASN_684, ASN_700, and GLU_701; PRO_221, GLY_275, ALA_487, and GLU_561; CYS_540, GLU_560, and SER_798; ILE_104, GLY_218, ARG_316, GLY_383, GLU_589, ASN_684, and SER_762; ASP_41, SER_258, ARG_316, ARG_512, VAL_609, and ASN_697; and GLY_10, ASP_160, VAL_184, GLU_277, GLY_383, TYR_546, VAL_609, GLY_698, GLU_740, ASP_761, GLU_800, and ASP_821; amino acids respectively. The corresponding average fluctuations of the wild and mutated proteins were 2.69 Å, 2.74 Å, 2.61 Å, 3.40 Å, 2.75 Å and 2.95 Å. The highest and lowest fluctuation values of wild type and I123S, V456M, D463G, R602H, and R633W proteins were calculated as 16.50 Å, 20.46 Å, 14.03 Å, 13.63 Å, 13.95 Å, 19.72 Å, and 0.94 Å, 1.00 Å, 0.75 Å, 0.95 Å, 0.80 Å, 0.88 Å respectively.

### Radius of gyration (R_g_)

The Rg quantifies the distribution of atoms around a protein axis, and serves as a crucial metric for forecasting macromolecular structural behavior and evaluating alterations in protein compactness. Here, the complex stability of the wild type and mutated proteins was assessed by analyzing their Rg values throughout a 50 ns simulation period. The Rg value of wild protein and five selected missense SNPs I123S, V456M, D463G, R602H, and R633W ranges from 35.946 to 42.364 Å, 35.617–39.260 Å, 35.898–42.184 Å, 36.455–39.407 Å, 35.740–39.254 Å and 35.828–43.499 Å, respectively (Fig. 5C). The average fluctuations of these SNPs were 37.235 Å, 36.824 Å, 37.280 Å, 37.889 Å, 36.836 Å and 38.037 Å, respectively. Unstable mutated protein structures in 50 ns simulations with a lower fluctuation range suggested that the binding affinity of the selected ligand did not significantly alter the active site of the corresponding protein.

### Hydrogen bonds

Hydrogen bonds are pivotal for ensuring the binding stability of the corresponding protein. The number of hydrogen bonds can define the protein characteristics, structural stability and ability to bind with other molecules. Therefore, the number of hydrogen bonds in the wild protein and five mutated proteins (I123S, V456M, D463G, R602H, and R633W) (Fig. 5D). All the proteins formed multiple hydrogen bonds ranging from 670 to 790 in 50 ns simulation time. Higher specificity and less flexibility of hydrogen bonds in wild protein compared to the mutated protein might be due to the higher structural instability of the mutated protein compared to WT (Fig. 5D).

### Gene–gene and protein–protein interaction networks

#### GeneMANIA

**Figure 6:**
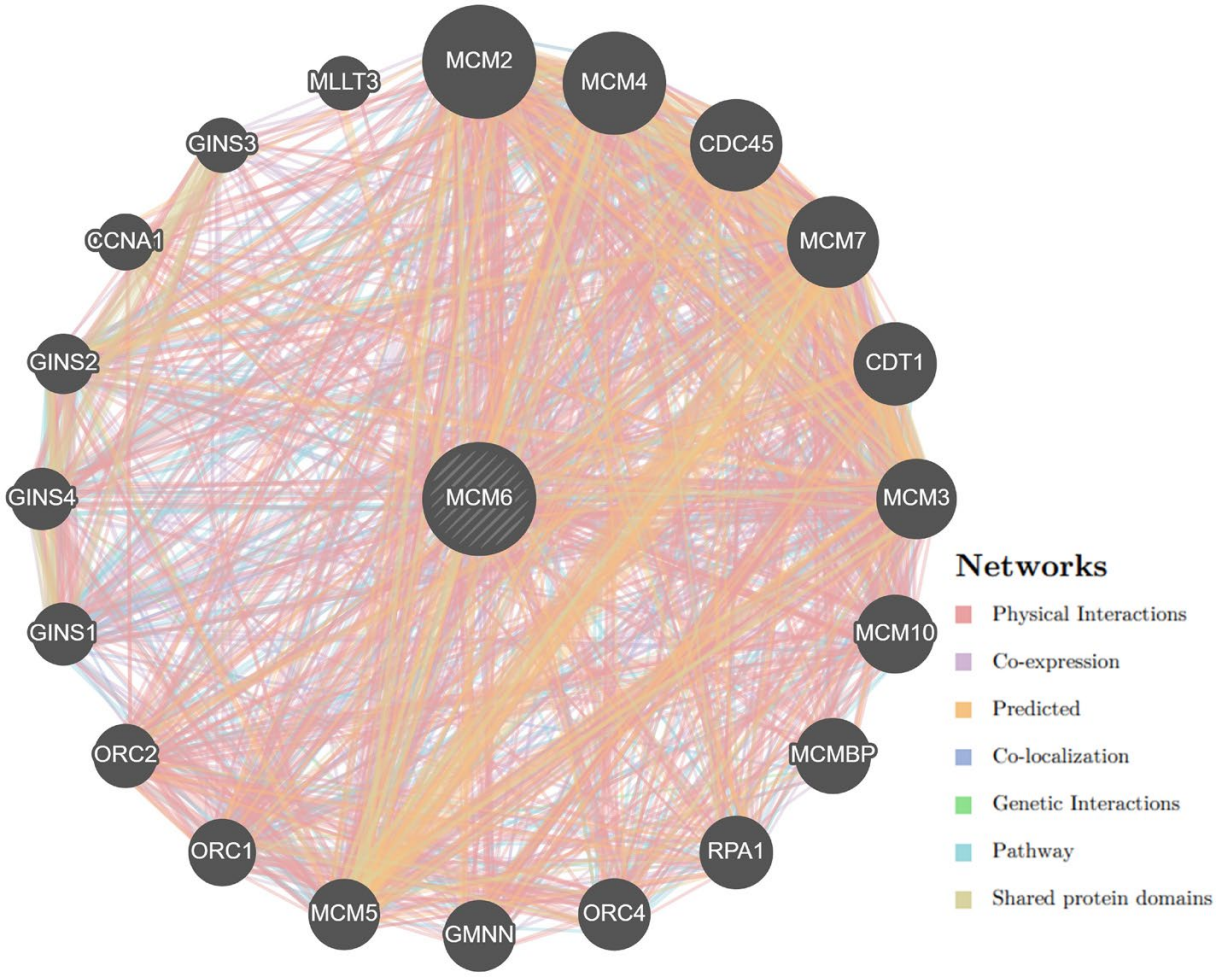
Gene–gene interaction network of *MCM6* gene predicted by GeneMANIA. GeneMANIA^52^ tool was utilized to generate the image (http://www.genemania.org).

GeneMANIA constructed a composite gene–gene functional interaction network for the *MCM6* gene (Fig. 5). The *MCM6* gene was found to be associated with 20 other genes which play vital roles in various functions. Among these 20 genes, the most important were the *MCM2, MCM4, CDC45, MCM7,* and *CDT1* genes (Fig. 6 and Supplementary Table S4).

#### STRING

**Figure 7:**
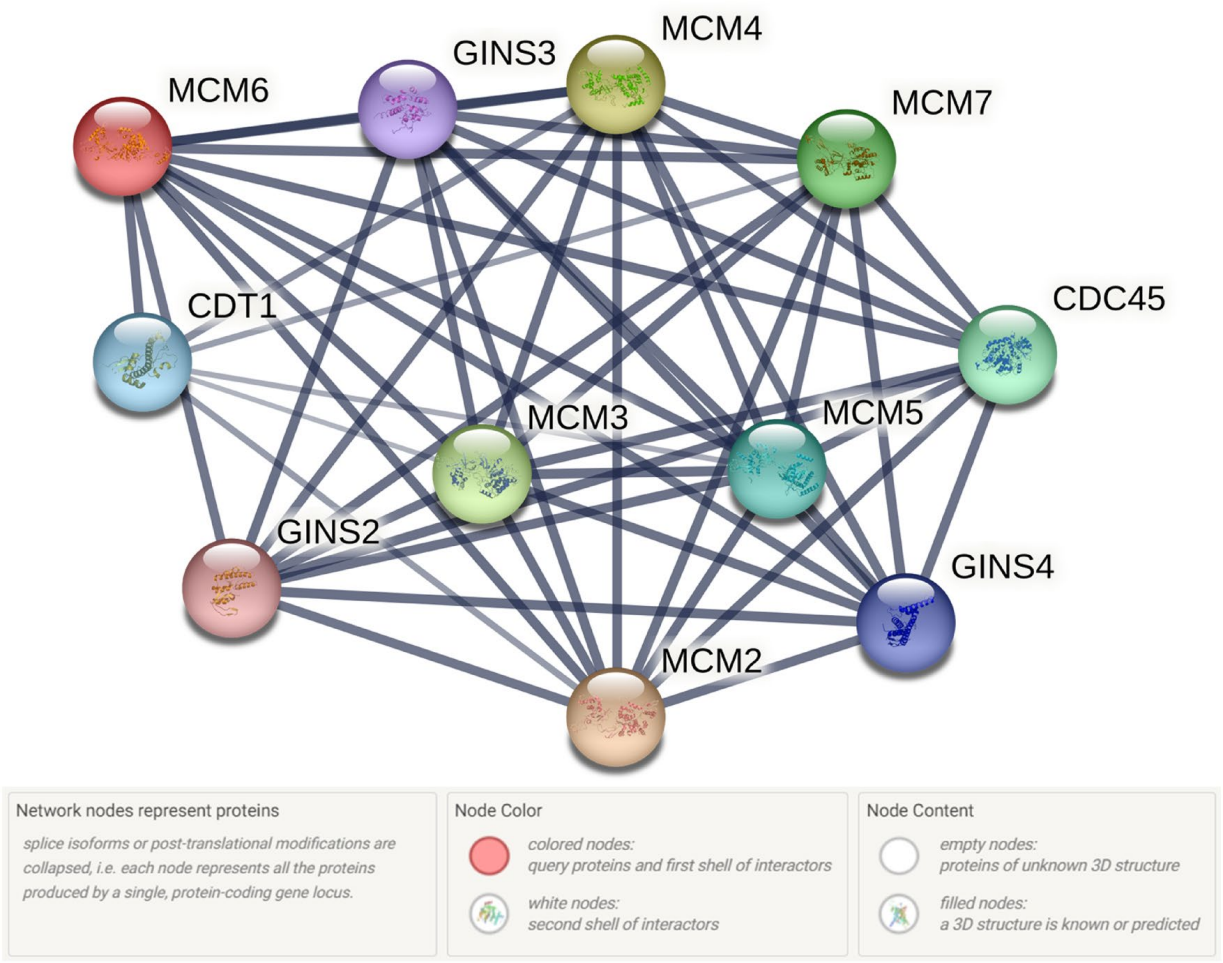
Interaction of MCM6 protein with other proteins analyzed by STRING tool.

The STRING database was used to partially describe the functional relationships and interaction networks of *MCM6* gene. The analysis revealed that *MCM6* gene was associated with 10 other genes. A significant correlation was observed between the topological characteristics and biological function of corresponding genes. Among these genes, *MCM2, MCM4, CDC45, MCM7, CDT1, MCM3, MCM5, GINS4, GINS2* and *GINS3* genes showed the strongest interactions with the corresponding gene (Fig. 7 and Supplementary Table S5)^55^.

## Discussion

The MCM family proteins are highly conserved hexameric complexes of DNA-binding proteins. There are six subtypes of MCM proteins, namely, MCM2, MCM3, MCM4, MCM5, MCM6 and MCM7^56^. Among these, the MCM6 protein is particularly important for cell proliferation and the regulation of DNA replication^14^. The *MCM6* gene, which encodes the MCM6 protein is found in the human genome^16^. Mutations in the *MCM6* gene can lead to lactose intolerance, lactose non-persistence and metabolically unhealthy obesity in children^57,58^. Several missense SNPs in the *MCM6* gene have been reported in the dbSNP database. To better understand the mechanism by which these mutations affect the structural integrity of proteins and contribute to disease pathogenesis, a systematic deep bioinformatics analyses have been conducted to identify functionally important missense SNPs in the corresponding gene. We used *in-silico* structural and functional analyses to identify potential missense SNPs, as well as various computational approaches to predict the deleterious SNPs.

A total of 642 missense SNPs among 15,009 SNPs in the analyzed gene indicate how the significant number of SNPs could alter the protein structure of the *MCM6* gene. A series of analyses of missense SNPs using SIFT, Polyphen-2, SNAP, PhD-SNP, PANTHER and SNPs&GO guides for precise screening of the most deleterious SNPs. Among the 33 deleterious SNPs identified by SIFT, 23 probably damaging SNPs revealed that all deleterious SNPs could not have potential for disease pathogenesis as they could alter protein function (Table 1). Variations in computing diseases associated with missense SNPs in SNPs&GO, PhD-SNP, and PANTHER might be due to using different algorithms in the mentioned programs. However, the consistent common 12 missense SNPs in all the analyzed programs might be due to their role in disease pathogenesis (Table 1). The eleven (I123S, R207C, R222C, L449F, V456M, D463G, H556Y, R602H, R633W, R658C, and P815T) which are deleterious, probably damaging, effective and disease associated missense SNPs in the *MCM6* gene guide the identification the molecular mechanism by which these SNPs cause disease pathogenesis altering protein function (Table 1). Then, highly functional R222C, R222C, D463G, R602H and R633W SNPs in the conserved region guide elucidating the molecular mechanisms by which these SNPs significantly alter protein function over several generations (Fig. 2). Mutations in highly conserved regions are more destructive than in non-conserved region^5^.

Protein stability plays a critical role in maintaining the biological functions and activities of biomolecules. Pathogenic missense mutations lead to incorrect folding and decreased stability of the altered protein. The significant protein destabilization potential of eight missense SNPs (I123S, R207C, R222C, V456M, D463G, R602H, R633W, and R658C) in the *MCM6* gene might be due to their significant role in protein bonding and folding. This destabilization might promote diseases pathogenesis. Six rigid SNPs (I123S, R207C, R222C, V456M, R602H, R633W) predicted by MEDUSA might reveal their potential to increase disease pathogenesis by increasing the rigidity of the mutant protein (Fig. 3A). Mutation-mediated overall flexibility decreases and rigidity increases might affect the binding affinity of the mutant proteins^59^. Amino acid substitution significantly alters the 3D structure and function of the corresponding protein^60^. Alpha helix and beta strands are the structural elements of a protein where the former one represents intramolecular hydrogen (H) bonding and the latter consists of beta sheets. Alpha helix and beta strand significantly differ in mutation tolerance^61^. Alpha helix has potential to accumulate more mutations due to the higher numbers of inter-residue contacts and mutations to residues in β-strands reduce the volume of the amino acid^62^. The 29% alpha-helix in the WT and 29–31% in the mutant, along with 22–24 disordered percentage reveals that the mutant proteins lacked a fixed ordered 3D structure (Fig. 3B and Supplementary Table S3). Mutations affect the helix–helix interactions and disordered regions of corresponding protein by changing their properties^63^. Hence, mutations at these positions might alter the structural and functional properties of the mutant protein.

MutPred2 mediated a significantly higher general score of > 0.75 in all 8 missense SNPs (I123S, R207C, R222C, V456M, D463G, R602H, R633W, and R658C) reveal that these mutations significantly alter the structural and functional properties of the corresponding proteins (Table 3). Since V456M and R633W mutants were larger and the rest were smaller than the WT, these mutations might significantly alter the functional properties of the proteins (Table 4). Mutation of the residue interrupts inter/intra-molecular interactions of the protein which influence the function, characteristics or reactivity of the mutant protein^64^. Therefore, mutations in the residues at the above-mentioned eight positions might alter the function, characteristics or reactivity of the WT protein. Significant destabilization potential in six mutations (I123S, R207C, V456M, D463G, R602H, and R633W) as analyzed by DynaMut2 might alter the structure of the mutant protein leading to loss of function (Table 5). The distribution of five (I123S, V456M, D463G, R602H, and R633W) mutations in MCM (PF00493) and MCM_N (PF14551) domains might be due to the significant contribution of these mutations to the functional alteration of the protein and progression of disease pathogenesis (Fig. 4). Domains are functionally active sites in a protein structure and mutations at these sites may have a tremendous effect on their activity^65^. Hence, the corresponding proteins of these five mutations might have a harmful effect at supra-optimal level in the human genome. The molecular dynamics simulation evaluates how missense SNPs affect the stability, residual fluctuation, and compactness of the protein at different levels. This guide identifies novel mutations in the corresponding protein in an effective way. The protein structure is stabilized when the RMSD and RMSF values of a protein are within 1–3 Å^66^. The consistent fluctuation of the I123S, V456M, D463G, R602H, and R633W mutant in RMSD suggests that these five mutations cause unstable structures of the corresponding protein (Fig. 5A). The RMSF evaluates the mean fluctuation of WT and mutant structures to determine the compactness of the protein. The higher fluctuation in mutants compared to WT was due to the structural instability of the corresponding protein (Fig. 5B). Compared to the WT, a higher Rg value represents the disassociation of the respective protein. However, the increased number of hydrogen bonds causes the structural unsteadiness^67,68^. Hence, consistent fluctuations in RMSD and RMSF values, high Rg and hydrogen bonds in mutant proteins compared to WT might guide exploring the mechanism by which these missense SNPs alter the structure and function of the native MCM6 protein. In the GeneMANIA mediated gene–gene functional interaction network, *MCM6 *significantly interacted with 20 others genes. Among these, *MCM2, MCM4, CDC45 (Cell division cycle 45), MCM7,* and *CDT1 (Chromatin licensing and DNA replication factor 1)* genes interacted more significantly than the other genes (Fig. 6 and Supplementary Table S4). *MCM2* regulates cell cycle and DNA replication-related pathways^8^, and while *MCM4* acts as the replicative helicase and is required for DNA replication and genome stability^69^. *CDC45* is essential for the establishment of an initiation complex at DNA origins^70^, *MCM7* is responsible for markedly increased DNA synthesis, cell proliferation and an increased cell invasion in prostate cancer^71^, and *CDT1* provides instructions for making a protein that is important in the copying of a cell's DNA before the cell divides^72^. Hence, missense SNPs in *MCM6* might alter DNA replication and cell proliferation by interacting with other 20 genes^73–84^ and resulting in serious health hazards even cancer.

In STRING based protein–protein interaction, the MCM6 protein significantly interacted with 10 different proteins^8,11,14,69–72,80,83,85,86^ having the confidence score of ≥ 0.9^87^ (Fig. 7 and Supplementary Table S5). Among these 10 proteins, eight common interacting proteins [MCM5, MCM4, MCM2, GINS4, CDT1, MCM7, GINS3 (GINS complex subunit 4), CDC45] in GeneMANIA and STRING based analyses indicate that MCM6 precisely interacted with the mentioned genes (Supplementary Tables S4 & S5). Almost consistent interactions result in GeneMANIA and STRING based analyses reveal that the missense SNPs in the *MCM6* gene might alter the structural and functional integrity of the gene along with interacting major genes involved in DNA replication and cell proliferation pathways.

In northern European populations, the MCM6 rs4988235 SNP (commonly referred to as LCT-13910 C/T) is highly correlated with lactase persistence^88,89^. The rs3754686 SNP in *MCM6* gene occurs more frequently globally^90^. Numerous studies have been conducted on the lactase persistence-associated genetic variants of the *MCM6* gene, including rs145946881, rs869051967, rs41380347, rs4988235 and rs41525747^91^. In children, rs1057031 contributes the most to the development of metabolically unhealthy obesity^92^. The situation is different in the Arabian Peninsula and East Africa, where four different mutations have been found to be associated with lactose persistence. These mutations include rs41525747, rs41380347, rs820486563, and rs145946881, all of which cluster in the *MCM6* gene^25,93, 94^.

Although different SNP in the *MCM6* gene that significantly progress to different disease pathogenies have already been reported, the molecular mechanism of how our identified most significant missense SNPs (I123S, V456M, D463G, R602H, and R633W) cause disease pathogenesis has not yet been discovered. The distribution of these five missense SNPs in the PF00493 and PF14551 domains, which are involved in DNA replication, cell division and cell proliferation reveals that these missense SNPs in the *MCM6* might alter their function. Mutated proteins might significantly contribute to the pathogenesis as MCM6 consistently interacted with different genes in the pathways involved in DNA replication and cell division as predicted by GeneMANIA and STRING. The results of the MD simulations also support the findings. Integrating the results of all our analyses on how missense SNPs of the *MCM6* gene alter its structural integrity and functional properties, programming based synthetic genetic circuit enabled personalized drugs could be innovated for individuals with missense SNPs of the *MCM6* gene in the mentioned positions. This requires a deep analysis of the mentioned missense SNPs along with the integration and application of synthetic biology, machine learning and artificial intelligence under in silico, in vitro, and in vivo conditions.

## Conclusion

Here, we identified 642 (4.28%) missense SNPs from 15,009 SNPs for the *MCM6* gene. Then, a series of precise bioinformatics analyses were performed to identify the deleterious, probably damaging, effective and disease-associated, highly harmful and destabilizing nsSNPs that can alter the structure and function of the MCM6 protein. After a series of analyses, 11 missense common SNPs (I123S, R207C, R222C, L449F, V456M, D463G, H556Y, R602H, R633W, R658C and P815T) were found to be deleterious, probably damaging, affective, and associated with diseases. Subsequently, 8 missense SNPs were found to be highly harmful and significantly contribute to disease pathogenesis. Finally, five mutations (I123S, V456M, D463G, R602H, and R633W) were found to be more harmful since those are located in two domains. Consistent fluctuations in RMSD and RMSF value and high Rg and hydrogen bond in mutant proteins compared to WT during MD simulations reveal that these mutations might alter the protein structure and stability of the WT protein and may have a significant contribution to disease pathogenesis. Considering the impact of these missense SNPs along with their interacting pathways, personalized medicine could be developed to mitigate the harmful effects of these missense SNPs on the diseased individual/population.

### Supplementary Information

Supplementary Table S1.

Supplementary Table S2.

Supplementary Table S3.

Supplementary Table S4.

Supplementary Table S5.

## Data Availability

All data are included in the manuscript.
