# Supplementary material for: In silico functional, structural and pathogenicity analysis of missense single nucleotide polymorphisms in human MCM6 gene
Source: Sci Rep. 2024 May 21;14:11607. doi: 10.1038/s41598-024-62299-2 (PMC11109216; doi:10.1038/s41598-024-62299-2)
Supplement: Supplementary file 1 — Supplementary Table S1. [file 41598_2024_62299_MOESM1_ESM.docx]

**Table S1.** Prediction of deleterious missense SNPs of *MCM6* gene using SIFT.

| **SL** | **SNP ID** | **Amino Acid Substitution** | **Prediction Score** | **SIFT Prediction** |
| --- | --- | --- | --- | --- |
| 1 | rs201423668 | S76F | 0.001 | Deleterious |
| 2 | rs61752701 | R92W | 0.003 | Deleterious |
| 3 | rs149573817 | A113T | 0.009 | Deleterious |
| 4 | rs201187605 | I123S | 0.001 | Deleterious |
| 5 | rs201824504 | R207C | 0.001 | Deleterious |
| 6 | rs144893830 | R222C | 0 | Deleterious |
| 7 | rs147531807 | Q237E | 0.014 | Deleterious |
| 8 | rs139876145 | T247I | 0.022 | Deleterious |
| 9 | rs374456478 | V250I | 0.005 | Deleterious |
| 10 | rs199894424 | S268F | 0.031 | Deleterious |
| 11 | rs200725312 | L284F | 0.003 | Deleterious |
| 12 | rs138593296 | R285W | 0 | Deleterious |
| 13 | rs144937311 | T357I | 0.006 | Deleterious |
| 14 | rs368811703 | T384N | 0.036 | Deleterious |
| 15 | rs113753889 | E438G | 0.001 | Deleterious |
| 16 | rs376760086 | I444M | 0 | Deleterious |
| 17 | rs375104649 | L449F | 0.003 | Deleterious |
| 18 | rs191517067 | V456M | 0.002 | Deleterious |
| 19 | rs199696245 | D463G | 0 | Deleterious |
| 20 | rs138808270 | R468W | 0.006 | Deleterious |
| 21 | rs377587920 | Q470R | 0 | Deleterious |
| 22 | rs201501566 | I482V | 0 | Deleterious |
| 23 | rs149081066 | H556Y | 0 | Deleterious |
| 24 | rs184578188 | R602H | 0 | Deleterious |
| 25 | rs374533979 | D605G | 0.002 | Deleterious |
| 26 | rs267598892 | R633W | 0 | Deleterious |
| 27 | rs55828049 | E647V | 0.019 | Deleterious |
| 28 | rs373818867 | I656V | 0.028 | Deleterious |
| 29 | rs142938887 | R658C | 0.002 | Deleterious |
| 30 | rs376497941 | G681C | 0.041 | Deleterious |
| 31 | rs140051424 | D688Y | 0.005 | Deleterious |
| 32 | rs200993395 | R732T | 0.009 | Deleterious |
| 33 | rs1804609 | P815T | 0 | Deleterious |
