# Supplementary material for: In silico functional, structural and pathogenicity analysis of missense single nucleotide polymorphisms in human MCM6 gene
Source: Sci Rep. 2024 May 21;14:11607. doi: 10.1038/s41598-024-62299-2 (PMC11109216; doi:10.1038/s41598-024-62299-2)
Supplement: Supplementary file 3 — Supplementary Table S3. [file 41598_2024_62299_MOESM3_ESM.docx]

**Table S3.** Characterization of three-dimensional modeling of the MCM6 protein and others 8 missense SNPs variant proteins.

| **Gene ID** | **Alpha helix**  **(%)** | **Beta strand (%)** | **Disordered**  **(%)** | **Confidence**  **(%)** | **Coverage**  **(%)** |
| --- | --- | --- | --- | --- | --- |
| MCM6_Wild Type | 29 | 18 | 24 | 100 | 83 |
| I123S | 29 | 18 | 24 |  |  |
| R207C | 29 | 18 | 23 |  |  |
| R222C | 29 | 18 | 23 |  |  |
| V456M | 30 | 17 | 22 |  |  |
| D463G | 30 | 17 | 24 |  |  |
| R602H | 30 | 17 | 24 |  |  |
| R633W | 31 | 18 | 24 |  |  |
| R658C | 29 | 18 | 24 |  |  |
