# Supplementary material for: In silico functional, structural and pathogenicity analysis of missense single nucleotide polymorphisms in human MCM6 gene
Source: Sci Rep. 2024 May 21;14:11607. doi: 10.1038/s41598-024-62299-2 (PMC11109216; doi:10.1038/s41598-024-62299-2)
Supplement: Supplementary file 4 — Supplementary Table S4. [file 41598_2024_62299_MOESM4_ESM.docx]

**Table S4**. Description of genes that significantly interacted with *MCM6* gene during gene-gene

interaction network as predicted by GeneMANIA.

| **Gene** | **Description** | **Function** |
| --- | --- | --- |
| MCM6 | Minichromosome maintenance complex component 6 | DNA replication regulator that plays a crucial role in sustaining the cell cycle^82^. |
| MCM2 | Minichromosome maintenance complex component 2 | Regulating cell cycle- and DNA replication-related pathways^69^. |
| MCM4 | Minichromosome maintenance complex component 4 | Acts as the replicative helicase and is required for DNA replication and genome stability^70^. |
| CDC45 | Cell division cycle 45 | Essential for establishment of an initiation complex at DNA origins^71^. |
| MCM7 | Minichromosome maintenance complex component 7 | Responsible for markedly increased DNA synthesis, cell proliferation and an increased cell invasion in prostate cancer^72^. |
| CDT1 | Chromatin licensing and DNA replication factor 1 | Provides instructions for making a protein that is important in the copying of a cell's DNA before the cell divides^73^. |
| MCM3 | Minichromosome maintenance complex component 3 | Responsible for replication licensing and is implicated in the formation of the replicative helicase during the replication progression^83^. |
| MCM10 | Minichromosome maintenance 10 replication initiation factors | Promotes the initiation of DNA replication through direct interactions with the cell division cycle 45 (Cdc45)^84^. |
| MCMBP | Minichromosome maintenance complex binding protein | Promotes the assembly of the MCM2-7 hetero-hexamer to ensure robust DNA replication in human cells^85^. |
| RPA1 | Replication protein A1 | DNA replication and repair and involved in telomere maintenance^86^. |
| ORC4 | Origin recognition complex subunit 4 | Fundamental function in the process of initiation of replication by remodeling the structure of origin of replication using the energy of supercoiled DNA^87^. |
| GMNN | Geminin DNA replication inhibitor | Maintenance helicases (MCM) loading onto the chromatin-bound origin recognition complex^88^. |
| MCM5 | Minichromosome maintenance complex component 5 | Involved in DNA replication and cell proliferation^89^. |
| ORC1 | Origin recognition complex subunit 1 | Involved in the copying of cell structures called centrosomes and centrioles^90^. |
| ORC2 | Origin recognition complex subunit 2 | Orc2 bound in a cell cycle‐regulated manner to heterochromatin and HP1 proteins^91^. |
| GINS1 | GINS complex subunit 1 | Transcription Factor E2F1 Enhances Hepatocellular Carcinoma Cell Proliferation and Stemness by Activating GINS1^92^. |
| GINS4 | GINS complex subunit 4 | Essential for the establishment of DNA replication forks and replisome progression^93^. |
| GINS2 | GINS complex subunit 2 | Regulates the proliferation and apoptosis of colon cancer cells through PTP4A1^94^. |
| CCNA1 | Cyclin A1 | Inhibits osteoporosis by suppressing transforming growth factor-beta (TGF-beta) pathway in osteoblasts^95^. |
| GINS3 | GINS complex subunit 3 | Essential for the initiation of DNA replication and replisome progression in eukaryotes^96^. |
| MLLT3 | MLLT3 super elongation complex subunit | Regulator of erythroid/megakaryocytic (E/Meg) lineage decisions^97^. |
