# Supplementary material for: In silico functional, structural and pathogenicity analysis of missense single nucleotide polymorphisms in human MCM6 gene
Source: Sci Rep. 2024 May 21;14:11607. doi: 10.1038/s41598-024-62299-2 (PMC11109216; doi:10.1038/s41598-024-62299-2)
Supplement: Supplementary file 5 — Supplementary Table S5. [file 41598_2024_62299_MOESM5_ESM.docx]

**Table S5**. Description of proteins that significantly interacted with MCM6 protein when

analyzed by STRING tool.

| **Gene** | **Description** | **Function** |
| --- | --- | --- |
| MCM6 | Minichromosome maintenance complex component 6 | DNA replication regulator that plays a crucial role in sustaining the cell cycle^82^. |
| CDC6 | Cell division cycle 6 | Cdc6 is required to activate Chk1 when replication forks are stalled^98^. |
| MCM5 | Minichromosome maintenance complex component 4 | Involved in DNA replication and cell proliferation^89^. |
| MCM4 | Minichromosome maintenance complex component 4 | Acts as the replicative helicase and is required for DNA replication and genome stability^70^. |
| MCM2 | Minichromosome maintenance complex component 2 | Regulating cell cycle- and DNA replication-related pathways^69^. |
| GINS4 | GINS complex subunit 4 | Essential for the establishment of DNA replication forks and replisome progression^93^. |
| CDT1 | Chromatin licensing and DNA replication factor 1 | Provides instructions for making a protein that is important in the copying of a cell's DNA before the cell divides^73^. |
| MCM7 | Minichromosome maintenance complex component 7 | Responsible for markedly increased DNA synthesis, cell proliferation and an increased cell invasion in prostate cancer^72^. |
| CDC7 | Cell division cycle 7 | Play pivotal roles in DNA replication and contribute also to other aspects of DNA metabolism such as DNA repair and recombination^99^. |
| GINS3 | GINS complex subunit 3 | Essential for the initiation of DNA replication and replisome progression in eukaryotes^96^. |
| CDC45 | Cell division cycle 45 | Essential for establishment of an initiation complex at DNA origins^71^. |
